# *Chenopodium Quinoa* and *Salvia Hispanica* Provide Immunonutritional Agonists to Ameliorate Hepatocarcinoma Severity under a High-Fat Diet

**DOI:** 10.3390/nu12071946

**Published:** 2020-06-30

**Authors:** Jose Moisés Laparra Llopis, Daniel Brown, Blanca Saiz

**Affiliations:** Madrid Institute for Advanced studies in Food (IMDEA Food). Ctra. Cantoblanco 8, 28049 Madrid, Spain; browndanibrown@gmail.com (D.B.); blancasafi@hotmail.com (B.S.)

**Keywords:** hepatocarcinoma, protease inhibitors, myeloid cells, macrophages, microbiota

## Abstract

Complex interactions between immunonutritional agonist and high fat intake (HFD), the immune system and finally gut microbiota are important determinants of hepatocarcinoma (HCC) severity. The ability of immunonutritional agonists to modulate major aspects such as liver innate immunity and inflammation and alterations in major lipids profile as well as gut microbiota during HCC development is poorly understood. ^1^H NMR has been employed to assess imbalances in saturated fatty acids, MUFA and PUFA, which were associated to variations in iron homeostasis. These effects were dependent on the botanical nature (*Chenopodium quinoa* vs. *Salvia hispanica* L.) of the compounds. The results showed that immunonutritional agonists’ promoted resistance to hepatocarcinogenesis under pro-tumorigenic inflammation reflected, at a different extent, in increased proportions of F4/80^+^ cells in injured livers as well as positive trends of accumulated immune mediators (CD68/CD206 ratio) in intestinal tissue. Administration of all immunonutritional agonists caused similar variations of fecal microbiota, towards a lower obesity-inducing potential than animals only fed a HFD. Modulation of *Firmicutes* to *Bacteroidetes* contents restored the induction of microbial metabolites to improve epithelial barrier function, showing an association with liver saturated fatty acids and the MUFA and PUFA fractions. Collectively, these data provide novel findings supporting beneficial immunometabolic effects targeting hepatocarcinogenesis, influencing innate immunity within the gut-liver axis, and providing novel insights into their immunomodulatory activity.

## 1. Introduction

A major challenge of therapeutics is to tailor personalized approaches. Nutrition is experiencing profound changes, from its classical perspective assuring the accessibility to nutrients towards more consideration of the well-known bioactive potential of nutrients. Thus, it is necessary to develop personalized treatments based in molecular and metabolic alterations, combining complementary nutrition and precision medicine. Characterization of food ingredients with a high nutritional value and biological activity based on life-sciences will help developing innovative products with added value to improve health status.

Overnutrition causes an associated metabolic stress to increase leading to impairment of inflammatory responses and immune outcomes in humans, including non-alcoholic fatty liver disease (NAFLD) [1]. Epidemiological data makes evident that a significant proportion (10–20%) of subjects with NAFLD will develop the severe variant of non-alcoholic steatohepatitis (NASH) with high liver related morbidity and mortality, part of which is due to the development of hepatocellular carcinoma (HCC) [2]. Dietary patterns (i.e., food supply and preferences) together with the increase prevalence of risk factors such as obesity have contributed to NAFLD pushing it up as the most common liver pathology worldwide. During disease development, while the host’s endogenous factors leading to liver dysfunction are difficult to influence, the environmental factors are predominant and addressable in a preventive or therapeutic intention. In this context, research interests have evolved from total calorie intake and consumption to defining food composition. The latter and its interaction with the immune system and finally their crosstalk with the host’s gut microbiota may be even more important determinants of intestinal and liver immunometabolic health. Along the same lines, recent findings provided evidence of how gut microbiota can have a significant effect in modulating lipid metabolism [3]. The association of changes in gut microbiota and its microbiome with the fatty acid profile is one example of how nutrition can alter the local microenvironment and brings together the complexity of the areas of nutrition, immunity and the microbiome.

Despite the importance of innate immunity and intestinal lymphoid cells shaping lipid homeostasis [4,5] and gut microbiota [4], the intricate relationship between these different players is not yet completely understood. Changes in both innate immune conditions and gut microbial activity appear to have a bidirectional link, worsening or improving the established microbial metabolic steady-state commensalism [6]. Previous research has demonstrated that intestinal microbiota significantly contributes to methionine levels and tissue polyamines [7], affecting glucose, lipid and energy homeostasis. Here, metabolic imbalances leading to key intermediary metabolites (i.e., lipid profile, transsulfuration pathways) [8,9] can be directly associated to the pathophysiological consequences of NAFLD. All these events can have important consequences for innate immune signaling as lipopolysaccharide (LPS), from intestinal bacterial translocation, and ligands for “Toll-like” receptor (TLR)-4 are acylated by saturated fatty acids [10]. Otherwise, cysteine thiols of the TLR4/MD2 complex aid bacterial LPS binding to the receptor [11], which contribute to impair liver inflammation during NAFLD progression to NASH. It is known that the initiation and execution of cell death can be regulated by various lipids, and hepatic biotransformation has been shown to be significantly influenced by gut-microbiota-derived metabolites [9]. In this scenario, intestinal microbiota and TLR4 activation have been demonstrated as key determinants of HCC progression in chemically-induced (i.e., diethylnitrosamine) models [12].

Previous to our research efforts, it has shown that administration of an immunonutritional active fraction from *Chenopodium quinoa* and *Salvia hispanica* significantly induced innate immune response(s), which results critical for a successful amelioration of the HCC severity in mice [13]. Studies devoted to establishing structural and functional characterization of the bioactive compounds revealed the presence of serine-type protease inhibitors within homologous complexes with a certain homology in the different crops [14,15]. All in all, immunonutritional bioactive ingredients were identified as glycoproteins with a *N*-terminal glucuronamide linkage in *S. hispanica*, while it was as glucosides in *C. quinoa* [14]. At molecular-level, proteome analyses on human-like macrophages shed light on immunometabolic changes, which showed biological correlation with TLR4 signaling [15]. Viewed in the context of understanding how tolerance and immunosuppression regulate immune responses, the use of immunonutritional compounds as coadjutant strategies, among other, can represent a path forward to help developing durable and long-lasting immune response(s). Overall, little is known about how immunonutritional ingredients from *C. quinoa* and *S. hispanica* influence the immunometabolic adaptation as well as variations in gut microbiota to a pro-obesity diet affecting immune control of hepatic tumor progression.

The objective of this study is to investigate the potential impact of immunonutritional TLR4 agonists administration on the severity and consequences of early HCC development under a high fat diet consumption within the so called “gut-liver axis”. Here, it is hypothesized that immunonutritional compounds from *C. quinoa* and *S. hispanica* contribute to improving innate immunity and metabolic events at earlier stages of HCC progression to prevent liver dysfunction and the microbial burden to the disease severity. It was found that immunonutritional intervention, to a different extent, significantly increased the hepatic proportion of F4/80^+^ macrophages. Cytokines associated with pro-tumor neutrophils signaling such as granulocyte monocyte-colony stimulated factor increased in collagen-dense tumors. The lipid profile and relevant intermediaries from transsulfuration pathways were clearly influenced reducing HCC severity. In this scenario, disruption of intestinal microbial homeostasis seemed to be affected at the microbiome level.

## 2. Material and Methods

### 2.1. Immunometabolically Active Fractions from Plant Seeds

Commercial samples of *Chenopodium quinoa* and *Salvia hispanica* L seeds were purchased from local supermarkets (Madrid, Spain) to be used in the experiments. A salt-soluble extract enriched in protease (serine-type) inhibitors (PIs) was obtained as previously described [15], with minor implementations. In brief, salt-soluble extracts from flours were thermally-treated (60 °C) for 30 min, followed by sequential centrifugation, 1st step (8000× *g*) to remove the precipitate and 2nd step (10,000× *g*) through a 30 kDa membrane (Amicon^®^). Clear filtrates containing PIs were filtered through 0.45 µm and lyophilized until use.

To ensure the exclusion of bacterial lipopolysaccharide (LPS) contamination, filtrates were analyzed by ESI-Ms after isolation and purification of the active compounds prior to be used in the animal experiments.

### 2.2. Mice and Hepatocarcinoma Induction

Male mice were obtained from “Centro de Investigaciones Biológicas (CIB-CSIC)” (Madrid, Spain). Animal experiments were carried out in strict accordance with the recommendations in the Guide for the Care and Use of Laboratory Animals of CSIC (Consejo Superior de Investigaciones Científicas), and the protocol was approved by its Ethic Committee and the regional government (Ethic code, Proex 220/17). Before inducing the hepatocarcinoma (HCC) [12,13], animals were randomly distributed into different groups (*n* = 6 per group) and fed a normal chow or high-fat diet (AIN93G mod. HF 43 kcal% fat, irradiated, Ssniff spezialdiäten gmbh) from one week before inducing the HCC: (1) received vehicle (w/o PIs) and (2–3) received PIs solutions (100 µL from a 1 mg/mL working solution) from the first diethynitrosoamine (DEN) injection. In C57Bl/6 mice at age of 6-week-old, HCC was induced by the combination of DEN (20 mg/kg, i.p.) given 3 weekly (3-times per week) injections, and 24 weekly (3-times per week) administrations of Thioacetamide (TAA) (saturated solution—0.5 mL/kg i.g., dissolved in PBS—dose equivalent to 80 mg/kg). Mice were sacrificed 8 weeks after the initial DEN injection.

Changes in body weight and food consumption were monitored every two days. After treatment, mice were sacrificed by cervical luxation. Whole blood samples were preserved in EDTA treated tubes (at room temperature) for analyses. Different sections (1 cm) of the liver and intestine were fixed in 4% paraformaldehyde, embedded in OCT (Thermo Scientific™), immersed in RNA later buffer (Qiagen, Germantown, MD 20874, USA), Krebs’s buffer or RIPA buffer and kept at −80 °C until analysis.

### 2.3. Histologic and Morphometric Evaluation

Liver sections (5 μm) were stained with haematoxylin-eosin staining [13]. The samples were analyzed with a Nikon Eclipse 90i microscope equipped with a Nikon DS-5Mc digital camera. Photos were analyzed with the Nis Elements software (Nikon Instruments Inc., Melville, NY, USA). The parameters analyzed included number of intrahepatic nodules of mononuclear cells and disorganization of the hepatic parenchyma.

### 2.4. H NMR Analysis of Liver Lipids

Analyses were performed at Centro de Investigaciones Biomédicas ‘Alberto Sols’ (IIB-CSIC, Madrid Spain). HR MAS spectra were acquired from a 11.7 Tesla Bruker Avance spectrometer operating at 500.113 MHz, at 4 °C and 5 KHz spinning rate. 1D 1H HR MAS spectra were acquired using Carr Purcell Meiboom Gill (CPMG) sequence with 2 s water presaturation, 144 ms echo time and 128 scans, data were collected into 32 K data point using a spectral width of 10 KH (20 ppm) and water presaturation during relaxation delay of 2 s and 1D sequence for diffusion measurement using stimulated echo using bipolar gradient pulses for diffusion (stebpgp1s1d) with big delta 200 ms, little delta 1,2 ms, sine shaped gradient followed by a 300 us delay for gradient recovery, 5 kHz spectral width, 32 K data point and 128 scans.

Quantification of metabolites detectable in the spectra was performed by measuring the area of the peaks by using MestReC software (Mestrelab Research, Santiago de Compostela, Spain); data were manually phased and baseline corrected; NMR spectra were referenced to the FA terminal—CH3 signal at δ, 0.89 ppm. The analysis of the metabolites was performed by selecting the following resonances [16]: methyls (-CH3) at 0.89 ppm (saturated FA chains), -CH_3_ at 0.96 ppm (*n*-3 EPA and DHA), acyl chains methylenes (CH_2_) *n* at 1.33 ppm, CH_2_-C-CO at 1.58 ppm, CH_2_C=C at 2.02 ppm, CH_2_CO other than DHA at 2.25 ppm, CH_2_CO of DHA at 2.33 ppm, =C-CH_2_-C=at 2.78 ppm, CH=CH- at 5.33 ppm.

### 2.5. Immunofluoresce Analyses

Liver (4 µm) sections were incubated with a blocking solution (1X PBS/5% normal serum/0.3% Triton™ X-100) for 60 min [13]. After blocking, samples were washed with PBS. Adequate fluorescent-tagged antibodies (CD74 and F4/80) (Biolegend) were diluted in the dilution buffer (1X PBS/1% BSA/0.3% Triton™ X-100) and pipetted onto the slides. Samples were incubated overnight. Subsequently, after 3 washes of 10 min each in PBS-Triton X-100 0.05% (*v*/*v*) liver sections were analyzed using an inverted fluorescence microscope Leica DM IL LED.

### 2.6. Measurement of Myeloperoxidase (MPO) Activity

MPO activity was measured in the supernatants of hepatic tissue homogenates as a marker for neutrophil infiltration [13]. Aliquots of supernatants (50 μL) were assayed in a reaction mixture that contained 110 μL PBS, 20 μL of 0.22 M NaH_2_PO_4_ (pH 5.4), 20 μL of 0.026% (*v*/*v*) H_2_O_2_, and 20 μL of 18mM tetramethylbenzidine in 8% (*v*/*v*) aqueous dimethylformamide. After 10 min of reaction at 37 °C, 30 μL sodium acetate (1.5 M; pH 3) was added, and the absorbance at 620 nm was read in a microtiter plate reader. The activity was expressed as mU/mg protein.

### 2.7. Microbiological Analyses

The composition of the microbiota was analyzed by real-time PCR ([17], Supplementary Table S1.) Samples of transversal colon (1 cm) were collected, diluted (0.5 mL) in PBS (pH 7.2) and homogenized thoroughly by agitation in a tissue lyser (Qiagen, Hilden, Germany). Aliquots were used for DNA extraction using the DNA stool Mini kit (NzyTech, Portugal) following the manufacturer’s instructions. Genus-, group- and species-specific primers were used as described previously to quantify the different bacterial groups of the intestinal microbiota.

### 2.8. Analysis of Bioactive Hepcidin and Microbial Short Chain Fatty Acids

Bioactive hepcidin was quantified in plasma samples using an Agilent HPLC system [18]. The column used in these analyses was a Poroshell C18 (50 × 2.7 mm) (Agilent, Madrid Spain). Briefly, the elution phases consisted of mobile phase A (0·125:1:500, trifluoroacetic acid–isopropanol–water) and mobile phase B (0·125:1:50:350:100, trifluoroacetic acid–isopropanol–water–methanol–acetonitrile). Aliquots (50 mL) of the precipitation supernatants were injected in each cycle, and the analysis was performed with the following gradient (min, %B): 0, 5; 30, 90; 33, 100; 35, 0; 40, 90; 45, 5.

Aliquots of colon samples were kept in 0.2 mL of 2N H_2_SO_4_ [19]. The samples were homogenized (1 min) using a TissueRuptor (Qiagen, Germantown, MD 20874, USA) and vortexed for 30 s. Afterwards, the mixtures were centrifuged (10,000 g, 10 min), and the supernatant was collected and diluted in deionized water prior to filtration (0·45µm, MillexGN; Millipore, Darmstadt, Germany). The quantification of organic acids was performed on a Poroshell C18 column (75 × 4.6 mm) (Agilent, Madrid Spain) using a 1290 Agilent HPLC system equipped with a multisolvent pump and a multivariable wavelength absorbance detector set at 214 nm. The elution was performed using 1% acetronitrile in 20 mM phosphate buffer adjusted to pH 2·20 with phosphoric acid (A) and water–acetronitrile (80:20, *v*/*v*, B) according to the following gradient: 0 min, 0% B; 5 min, 0% B; 12 min, 10% B; 19 min, 10% B.

### 2.9. Statistical Analyses

Statistical analyses were performed using SPSS v.15 software (SPSS Inc., Chicago, IL, USA). For normally distributed data, ANOVA with the post hoc Tukey test was applied, and for non-normally distributed data from microbial analyses, the Mann–Whitney U test was used [17]. Statistical significance was established at *p* < 0.05 for all comparisons.

## 3. Results and Discussion

### 3.1. Potential Role of Protease Inhibitors to Attenuate Liver Injury

**Figure 1:**
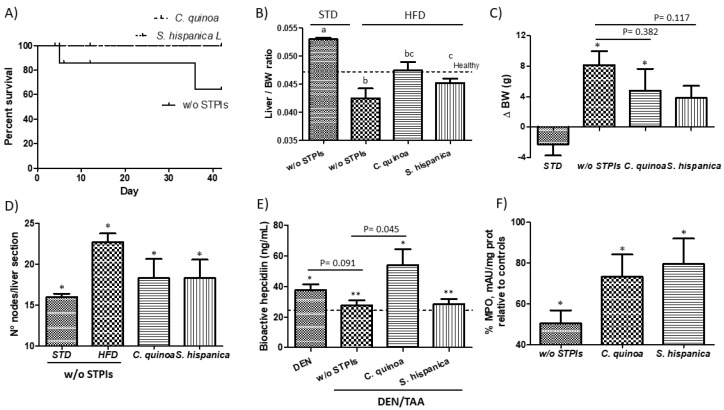
Hepatocarcinoma (HCC) development by the concurrent diethylnitrosamine (DEN) injection and thioacetamide (TAA) administration to 6 weeks old C57BL/6 mice. (**A**) Impact of protease inhibitors (PIs) in percent survival of HCC developing mice. (**B**) Changes in liver to body weight (BW) ratio and (**C**) Variations in body weight gain of high fat diet-fed animals receiving PIs. (**D**) Influence of feeding PIs in the intrahepatic accumulation of nodes of mononuclear cells in DEN/TAA-treated mice administered with PIs. (**E**) Serum levels of bioactive hepcidin peptide concentration. (**F**) Hepatic changes in myeloperoxidase activity (MPO) in liver samples of HCC developing mice. * Indicates statistical differences (Tukey–Kramer’s test, *p* < 0.05) to DEN/TAA-treated animals not receiving PIs.

In HCC developing mice fed a high-fat diet (HFD), the administration of immunonutritional PIs either from *C. quinoa* or *S. hispanica* decreased mortality (Figure 1A). Of the mice that completed the study period, those under an HFD displayed a significantly lower hepatosomatic (liver/body weight) ratios than those fed a standard chow (STD) as well as untreated (healthy) controls (Figure 1B). The liver/body weight ratio (LBWr) of animals fed with PIs from *C. quinoa* was significantly increased in comparison to animals not receiving PIs. These values appeared normalized in relation to those calculated for untreated (healthy) animals. Feeding PIs to HCC developing mice did not cause significant variations in liver mass but reduced HFD-induced body weight gain (Figure 1C). The observed resistance to HFD-induced BW gain was not due to reduced food intake. In fact, HFD-fed mice administered with PIs showed significantly increased (by 45%) food intake.

Feeding the HFD exacerbated liver injury during HCC development, which was reflected in the increased number of intrahepatic nodules of mononuclear cells that was determined via histopathology (Figure 1D). This observation seems to support an enhanced tumorigenicity and the negative impact of HFD altering the hepatic immunometabolic function. Either lipotoxic insults derived from hepatic oxidative stress [20] and other alterations in lipid homeostasis (i.e., CD36) [21] have been identified as negative contributors worsening hepatic immunometabolic dysfunction to empower liver injury and tumor microenvironment. When comparing the incidence of intrahepatic nodules to that in animals receiving PIs, immunonutritional intervention reduced the tumor burden decreasing liver injury to favor nutrient homeostasis and survival.

Accumulating evidence indicates that tumor elimination at early stages of development may be more dependent on tumor phenotype than on origin [22]. Thus, given the previously established intricate link between lipid and hepatic iron homeostasis [23,24] and their link to cancer [21,25], it was examined how the production of bioactive hepcidin compares in HCC developing mice receiving PIs (Figure 1E). Administration of TAA to DEN-treated animals caused a downregulated plasmatic bioactive hepcidin production. This effect is suggestive of increased hypoxia conditions after TAA biotransformation to acetamide and TAA-S-oxide. Only animals receiving PIs from *C. quinoa* showed a significant increase in plasmatic bioactive hepcidin concentrations evidencing different features of the PIs from different botanical origin. These differences may, at least in part, shape cell interactions within the tumor microenvironment. Overall, these results suggest that PIs administration could mediate cellular sensitization to changes in cellular iron homeostasis. Increased plasmatic bioactive hepcidin concentrations in animals administered with PIs could be associated to a reduced proliferative Ras/RAF and nutrient-dependent mTOR signaling [26,27].

Changes in the hypoxia-regulated molecular mechanisms are associated to contrasting properties of polymorphonuclear neutrophils (NTs) in different tumor settings [28]. Here, a decreased hepatic MPO activity was observed in DEN/TAA-treated mice in relation to untreated (healthy) animals (Figure 1F). This behavior makes evident the loss of recruitment of NTs that may contribute to HCC progression. Besides, animals administered with PIs displayed higher hepatic MPO activity indicative of increased recruiting of NTs. A higher hepatic neutrophil population could impair hepatocytes insulin resistance, helping to increase the hepatic macrophage infiltration (Figure 1D). This effect is consistent with previous data supporting a positive effect of intrahepatic NTs in reducing tumor progression by increasing immune cell activation [13]. Similarly, it has been concluded there is a beneficial role of NTs suppressing fibrosis in the CCl_4_-induced chronic liver injury model [29]. This effect was attributed to execution of the antifibrotic function of bone marrow-derived macrophages. However, the NTs role still appears controversial, and it is accepted they have negative effects exacerbating acute liver injury [30].

**Figure 2:**
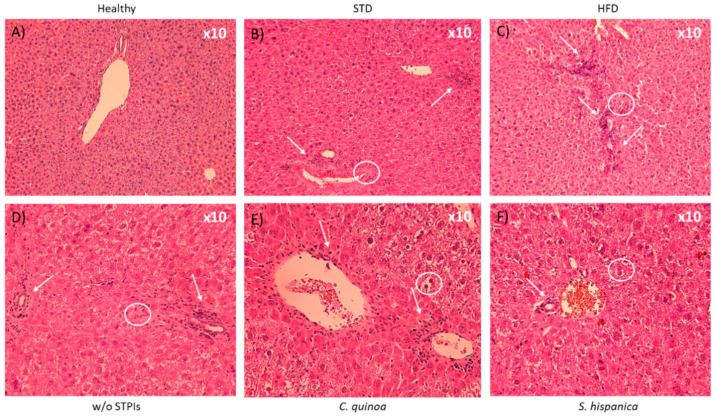
Liver hematoxylin and eosin staining of 14 weeks-old high fat diet-fed C57BL/6 mice administered with or without PIs (100 µg/animal) (**A**,**B**). At the end of the study period in the w/o STPIs group, 4 animals were analyzed, while in those administered with *C. quinoa* and *S. hispanica* 6 animals were analyzed. Arrows indicate prominent accumulation of nodes of mononuclear cells after 14 weeks. Circles locate irregular contours of the bile duct with nuclear pseudostratification and vacuolization of the epithelium, with marked portal inflammation (**C**,**D**). (**E**,**F**) Artifice that produces steatosis suggestive image.

Despite improved survival percentages of animals, which are consistent with previous studies [13], repeated doses of the extracts enriched in PIs were unable to abolish the chemically induced hepatocellular carcinoma (HCC) development. It was found after microscopic examination of H&E staining (Figure 2) that HFD increased periportal infiltrates and more pronounced ductal proliferation (Figure 2A–C). These observations were accompanied of acidophilic bodies and mitotic figures. Liver injury produced alterations that were reflected in perivascular infiltration of mononuclear cells (arrows) as well as distension of the vasculature (circles). These changes appear during early alterations in the liver’s normal role in lipid metabolism without development of fat vacuoles and signs of “ballooning”.

Taken together, these results suggest that promotion phase (i.e., TAA induced hepatic fibrosis) rather than initiation (i.e., DEN induced structural DNA changes) is the major interfering step for immunonutritional PIs from *C. quinoa* and *S. hispanica*. With the administration of repeated doses of PIs, all the histopathological markers were not abolished but became significantly less evident (Figure 2D–F). Collectively, these results indicated a remodeling and improvement process to reduce TAA-induced inflammation, which allows hypothesizing a reduced severity of fibrotic processes and tumor burden.

### 3.2. Pattern Changes in Lipid Profile

**Figure 3:**
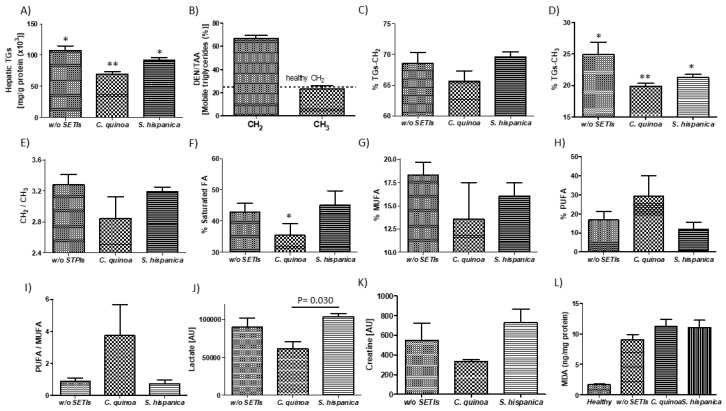
^1^H-MAS MR analyses of DEN/TAA-treated mice fed high fat diet administered with or without protease (serine-type) inhibitors (PIs) (100 µg/animal). (**A**) Colorimetric determination of total hepatic triglyceride content. ^1^H-MAS MR levels of triglyceride-CH_2_ (**B**) and triglyceride-CH_3_ (**C**) in tissue from HCC developing mice. (**D**–**F**) Lipid patterns for saturated fatty acids, MUFA and PUFA. (**G**–**I**) ^1^H-MAS MR levels of lactate (**J**), creatinine (**K**). Quantification of the oxidation marker malonaldehyde (MDA) in liver samples of HCC developing mice (**L**). Results are expressed as mean ± SEM (*n* = 4−6). * Indicates statistical differences (Tukey–Kramer’s test, *p* < 0.05) to DEN/TAA-treated animals not receiving PIs.

Cohorts of DEN/TAA-treated mice and PIs-administered mice, all fed an HFD, were assessed after the study period for changes in major lipid parameters (Figure 3). HCC developing mice exhibited higher hepatic triglycerides concentrations (Figure 3A) than the physiological values (150 mg/g protein) that are genetically determined in C57Bl/6 mice [31]. This result is concordant with the TAA (75 mg/kg, three times per week)-induced boosting of hepatic steato-hepatitic changes in livers of C57BL/6J mice fed with high-fat food diet [32]. As expected, a significant increase (by 40%) was quantified in the average presence of positive triglyceride-CH_2_ resonances (Figure 3B). The latter confirm the detection and presence of cell malignancy or malignant transformation (i.e., cell proliferation, necrosis, apoptosis, hypoxia and drug resistance) [16] reviewed in [33]. The sharp increase in mobile triglyceride-CH_2_ compounds is concordant with the important increase of major diglycerides (i.e., 34:1DG-a and 36:2DG), while cholesterol (Ch) and cholesterolester (ChE) were significantly diminished in STAM mice, which exhibit progression from NASH to fibrosis closely mimicking disease development in humans [34]. Otherwise, the relative abundance of triglyceride-CH_2_ and -CH_3_ molecules was significantly lower in 14-week-old HFD-fed mice when those were administered with PIs (Figure 3C,D). Thus, mice receiving repeated doses of PIs displayed significantly reduced triglyceride-CH_2_/-CH_3_ ratio supporting a decreased tumor burden phenotype (Figure 3E).

^1^H-MR analyses revealed further differences in major lipids profile with an average hepatic composition of 43% for saturated lipids, 15% for MUFA and 17% for PUFA (Figure 3F–H). The administration of PIs from *C. quinoa* trend to diminish the mean value of MUFA (by 5%) but significantly increased that of PUFA (by 13%) relative to animals not receiving PIs. Mice receiving *S. hispanica* only exhibited modest variations in MUFA as well as PUFA, which were opposite to those in animals administered with *C. quinoa*. Previous research efforts have shown that hepatic saturated fatty acid accumulation is causally associated to chronic liver disease and hint at immune functional connections between saturated fatty acids and impaired macrophage function and cell death [35]. Given the previously established role of dietary MUFA and PUFA to determine cell susceptibility to ferroptosis [36], the PUFA/MUFA ratio in hepatic samples was calculated (Figure 3I). Only animals receiving *C. quinoa* displayed a significant increased PUFA/MUFA ratio, in accordance with the plasmatic levels of bioactive hepcidin (Figure 1E) and the decreased triglyceride-CH_2_/CH_3_ values. Collectively, these data allow us to hypothesize the implication of iron homeostasis in controlling lipid management after immunonutritional intervention to reduce tumor burden.

**Figure 4:**
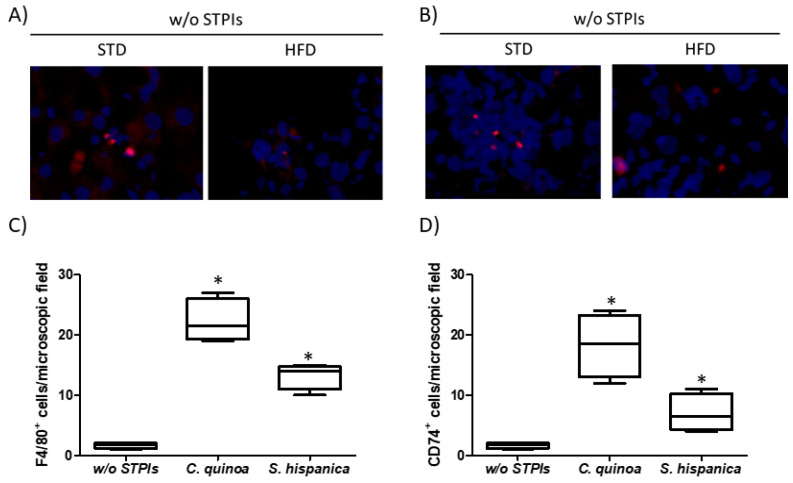
Immunological changes in hepatocarcinoma-developing mice administered with protease (serine-type) inhibitors (PIs). Number of hepatic single cells stained for (**A**,**C**) F4/80 and (**B**,**D**) CD74 (MHC-II invariant chain) quantified by immunofluorescence analysis. Cell counting was performed in 5 to 10 different microscopic fields from each of the stained sections per group. Results are expressed as mean ± SEM (*n* = 4−6). * Indicates statistical differences (Tukey–Kramer’s test, *p* < 0.05) to DEN/TAA-treated animals not receiving STPIs.

As PIs administration has been previously shown to induce changes in the immunological features of tumor developing injured livers [13], to gain insight into their potential when lipid homeostasis is altered, the variation of hepatic F4/80^+^ and CD74^+^ cells was evaluated (Figure 4). Immunofluorescence analyses on hepatic tissues from HFD fed animals confirmed the immunosuppressive environment that is reflected by the reduced F4/80^+^ and CD74^+^ cells, impairing early HCC control (Figure 4A,B). Administration of immunonutritional PIs helps to increase the hepatic infiltration of peripheral F4/80^+^ cells (Figure 4C), to a higher extent in those animals receiving PIs from *C. quinoa*. While increasing evidence suggests that CD74 is implicated in the biology of hepatocytes [37], it is unclear the role that it might play in cancer. These findings together with significant changes in major lipids profile (Figure 1C) helps to interpret the experimental data as decreased liver injury and hepatocyte transformation. Thus, it is hypothesized that at early stages of HCC development the immunonutritional-mediated CD74 overexpression could facilitate neutrophil recruitment [38] ameliorating the inflammatory milieu in injured livers. It is unknown whether it facilitated the control of proinflammatory processes synergized by the significantly increased average of PUFA (Figure 3H). Consistent with this, diminished PUFA levels have been quantified in the HFD fed STAM mice NASH model [34].

### 3.3. Hepatic Metabolic Intermediary Mediators in Inflammatory Perturbations are Reduced in Animals Receiving PIs

**Figure 5:**
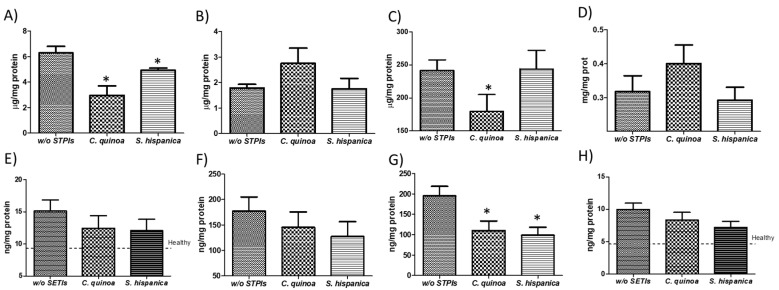
Hepatic concentration of low molecular weight thiols and cytokine profile in C57BL/6 wild-type mouse livers at 8 weeks from the initial concurrent diethylnitrosamine (DEN) injection and thioacetamide (TAA) administration. Tissue thiol concentration: homocysteine (**A**), N-acetyl cysteine (**B**), Cysteinyl-glycine (**C**) and Cysteine (**D**). Cytokine concentration: (**E**) Tumor necrosis factor (TNF)-α, (**F**) interleukine (IL)-6, (**G**) IL-17 and (**H**) granulocyte-monocyte colony stimulating factor (GM-CSF). Results are expressed as mean ± SEM (*n* = 4−6). * Indicates statistical differences (Tukey–Kramer’s test, *p* < 0.05) to DEN/TAA-treated animals not receiving PIs.

Decreased hepatocarcinogenic effects derived from M1-like polarization of the infiltrated hepatic macrophages is concordant with the reduced tumor-induced lactate levels (Figure 3J). Depletion of lactate can have important consequences for tumor progression as its accumulation in solid tumors is a pivotal and early event in the development of malignancies [39]. Here, immunosuppressive effects derived from lactate-induced M2-like polarization of tumor-associated macrophages [40] only appear manifest in animals not receiving PIs. However, no differences were seen for lactate between HFD fed animals receiving PIs from *S. hispanica* or those from the untreated group. These data suggest that interference with tumor progression cannot be completely attributed to the improvement of hepatic immunity and is likely due to PIs-induced innate immune signals that stem at intestinal level. Given the association of hepatic creatinine levels with the severity of lipotoxic insults due to hyperammonemia in in acute liver failure and endoplasmic reticulum stress-mediated hepatocellular apoptosis [41], intermediary metabolites of transsulfuration pathways in these animals were quantified (Figure 5).

Animals receiving PIs from *C. quinoa* displayed pattern changes in creatine levels consistent with an increased utilization of it, which was reflected in a significant reduction of the hepatic production of homocysteine (HCys) (Figure 5A). These changes could help explaining the diminished triglycerides accumulation [42,43] in the different groups of treatment (Figure 3A). The decreased concentrations of HCys are interpreted as positive effects reducing hepatocytes transformation via 4-epoxyeicosatrienoic acid production (i.e., CYP450 metabolism). These findings are consistent with changes in hepatic triglyceride-CH_2_/CH_3_ ratios (Figure 3C), supporting a more preserved hepatic cells plasma membrane triglyceride mass accumulation to regulate macrophages and neutrophils activation [44,45]. When monitoring changes in other intermediary metabolites within transsulphuration pathways, the positive trends in Cys-gly and Cys implicate the efficacy of PIs in the amelioration of DEN/TAA induced hepatocarcinoma. These results are concordant with an improved antitumoral response of interferon-α [46], which occurs via priming on TLR4-induced activation [47].

Administration of PIs had a positive impact on hepatic pro-inflammatory environment in HFD-fed animals, where cytokine and chemokine levels were decreased following PIs administration to HCC developing mice (Figure 5F–I). HFD favors structural and pattern changes within injured livers, which PIs administration seemed to diminish accompanied with an array of inflammation responses that slow-down the progression of hepatic injury in mice [48,49]. Besides, the cytokine and chemokine patterns do not fit those defined for M1-like (F4/80^+^) macrophage recruitment [50]. This behavior supports the macrophage polarization in distant organs (i.e., intestine). These results are concordant with the key role for liver mononuclear cells in the regulation of liver lipid metabolism previously reported [51].

### 3.4. Improvement in HCC Severity is Unlikely due to Changes in Gut Microbiota

**Figure 6:**
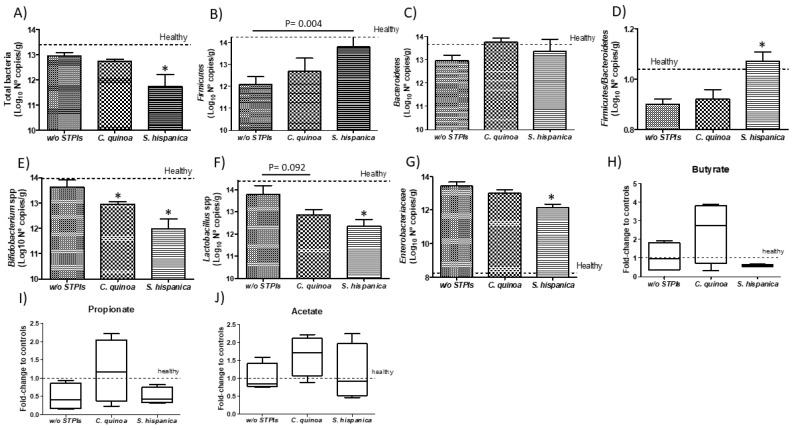
Colonic account of different bacterial phyla (**A**–**G**) of hepatocarcinoma developing mice administered with protease (serine-type) inhibitors (PIs). Relative variation of microbial metabolites (**H**–**J**). Results are expressed as mean ± SEM (*n* = 4−6). * Indicates statistical differences (Dunn’s test, *p* < 0.05).

Diethylnitrosamine (DEN)-induced pro-tumorigenic inflammation is recognized to cause profound changes in gut microbiota [52]. It was determined whether these alterations in gut microbiota are extended for DEN/TAA-induced hepatocarcinogenesis in animals under HFD receiving PIs (Figure 6). Major bacterial changes rely on the reduction of bacterial diversity, which is majorly reflected in changes in the *Firmicutes*/*Bacteroidetes* ratio, in relation to those values in healthy animals. These changes include genus of potential beneficial bacteria also accompanied of increased proportions of counts for the *Enterobacteriaceae* family (Figure 6A–G). These alterations in gut homeostasis are consistent with those previously reported for DEN-induced hepatocarcinogenesis [52]. These results showed that altered liver function impairs cross-feeding mechanisms of benign microbiota contributing to maintaining immune inflammation in the gut. The administration of immunonutritional PIs did not restore the numbers of total bacteria but aggravated bacterial loss to a different extent according to their origin (Figure 6A). Animals receiving *S. hispanica* exhibited most significant decreases in the proportion of total bacteria in comparison to *C. quinoa*. Major phylum as *Firmicutes* and *Bacteroidetes* displayed opposite growth rates in animals administered with *C. quinoa* or *S. hispanica* (Figure 6B,C). Thus, only animals administered with PIs from *S. hispanica* exhibited a relative more normalized variation between those major phyla, *Firmicutes* to *Bacteroidetes* ratio, in comparison to values calculated for untreated “healthy” animals.

Modulation of *Firmicutes* to *Bacteroidetes* contents, which potentially are reflected in functional outcomes of their microbiomes by PIs administration showed an association with liver saturated fatty acids and the MUFA and PUFA fractions (Figure 3D–F). These data suggest that fecal microbiota in HFD-fed animals receiving PIs displays lower obesity-inducing potential than that in mice not receiving PIs. Thus, it cannot be assumed that the negative impact of hepatocarcinoma on *Firmicutes*, which hosts beneficial *Clostridium* spp that function to temper expression of CD36 and lipid absorption [53], may contribute to aggravate HCC progression. Preclinical studies have provided contrasting results about the association of gut microbiota to a systematic shift from saturated or PUFA to MUFA lipids [3,9]. These authors stated a direct relation of liver fatty acid levels to gut microbial short chain fatty acid (i.e., acetate) producers. Along with decreased numbers of potential beneficial bacteria such as *Bifidobacterium* spp. and *Lactobacillus* spp., a significant decrease was quantified in members of the family *Enterobacteriaceae* (Figure 6G). In any case, the presence of potential pathogens such as *E. coli* or *B. fragilis* was detected. Here, such changes in SCFA production (i.e., butyrate and propionate) appeared most favored by changes in bacterial activity after administration of PIs from *C. quinoa* (Figure 6H–J). These microbiota derived metabolites are recognized to enhance intestinal hypoxia providing a signaling axis through hypoxia inducible factor (HIF)-1 to augment epithelial barrier and influence tissue function [54].

**Figure 7:**
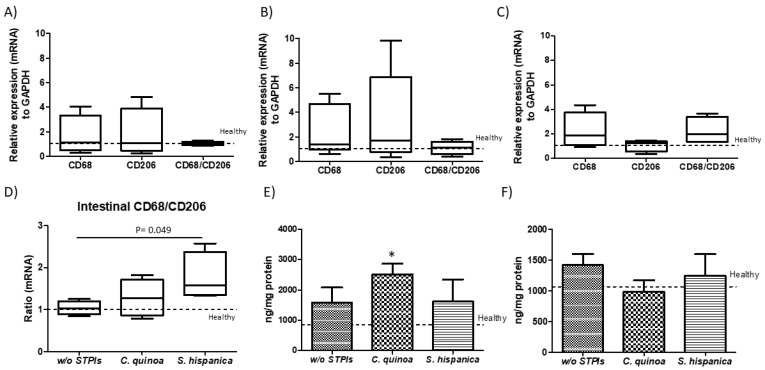
Immune mediators in duodenal sections from diethylnitrosamine (DEN) and thioacetamide (TAA)-treated mice compared to those administered with protease (serine-type) inhibitors (PIs) (100 µg/animal). Duodenal relative expression (mRNA) of the macrophage transmembrane glycoprotein CD68 and mannose receptor CD206 in animals administered without (**A**) or with PIs from *C. quinoa* (**B**) and *S. hispanica* (**C**) and comparative CD68/CD206 variations (**D**). Cytokine concentration: (**E**) Tumor necrosis factor (TNF)-α and (**F**) granulocyte-monocyte colony stimulating factor (GM-CSF). Results are expressed as mean ± SEM (*n* = 4−6). *Indicates statistical differences (Tukey–Kramer’s test, *p* < 0.05) to DEN/TAA-treated animals not receiving PIs.

Imbalances in the proportion of defined members within gut microbiota have been shown as critical for the metabolic capacities of the existing microbial communities [55]. Accumulated literature supports innate immunity as a determinant factor shaping gut microbiota and lipid homeostasis [4,5]. Expansion of *Enterobacteriaceae* can be caused by DEN/TAA-induced losses of immune regulatory mediators on gut homeostasis. However, the latter do not appear mediated by imbalances in beneficial *Bifidobacterium* spp. and *Lactobacillus* spp., whose metabolic capacities allow controlling *E. coli* overgrowth, supporting the influence of innate immunonutritional PIs on gut microbiota’s activity. Likewise, the resistance to hepatocarcinogenesis in DEN/TAA-induced pro-tumorigenic inflammation is thought to be afforded by administration of PIs promoting protection of innate immune mediators (Figure 7). Bacterial imbalances were reflected in a positive trend in the macrophage M1 versus M2 population at intestinal level in animals receiving PIs (Figure 7A–D). Only animals receiving *S. hispanica* displayed a significant increase in M1/M2 ratio (Figure 7E). These changes were accompanied of different variations for TNFα and GM-CSF concentrations (Figure 7E,F) in tissue samples from animals receiving *C. quinoa* and *S. hispanica*. Changes in the macrophage phenotypic adaptation cannot be attributed to the variation in the concentration of proinflammatory TNFα and GM-CSF, which in response to soluble CD14 released from monocytes as a marker of bacterial infections promote macrophages shift towards an anti-inflammatory phenotype [56].

## 4. Conclusions

This work reinforces the role of immunonutritional PIs as part of coadjutant strategies to pharmacological approaches favoring tumor control at early stages of development in the hepatic tissue. The statistical power of the study may be influenced due to survival percentage in the group not receiving PIs. However, the fact that the administration of PIs of *C. quinoa* and *S. hispanica* prevents animal death supports the reduction in the severity of HCC. For a number of reasons, it is likely that PIs administration drives immunometabolic adaptations reducing HCC severity in HFD-fed animals. PIs administration is associated to regulation in key nutrients homeostasis, activity of growth factors and increase in the proportions of infiltrated myeloid cells. Otherwise, administration of PIs did not re-establish major alterations in fecal microbial composition but appears to promote positive effects of intestinal innate immune modulation on functional outcomes from its microbiome. The interactions within the gut-liver axis provide significant insight into how immunonutritional PIs ultimately influence microbial activity and tissue function. These effects could be grounded in the crosstalk for induction of innate immune signaling in the intestinal lamina propria. Collectively, these data provide novel findings supporting beneficial immunometabolic effects of PIs from *C. quinoa* targeting hepatocarcinogenesis severity in HFD fed animals. These effects appear to be mediated influencing innate immunity within the gut-liver axis, providing novel insights into their immunomodulatory activity.

## Supplementary Materials

The following are available online at https://www.mdpi.com/2072-6643/12/7/1946/s1.
