# Supplementary material for: Chenopodium Quinoa and Salvia Hispanica Provide Immunonutritional Agonists to Ameliorate Hepatocarcinoma Severity under a High-Fat Diet
Source: Nutrients. 2020 Jun 30;12(7):1946. doi: 10.3390/nu12071946 (PMC7400258; doi:10.3390/nu12071946)
Supplement: Supplementary file 1 [file nutrients-12-01946-s001.pdf]

Supplementary Table 1. Genus-, group- and species-specific primers used to quantify the different bacterial groups of the intestinal microbiota.

Target bacteria	Primer	Sequence (5 to 3)	Product size (bp)	Annealing Tª (ºC)
Total bacteria	HDA1	ATCCCTACGGGAGGCAGCAGT	200	59
	HDA2	GTATTACCGCGGCTGCTGGCAC		
Lactobacillus	LAC1	ACGAGTAGGGAATCTTCCA	340	61
	LAC2	ATTYCACCGCTACACATG		
Bifidobacteria	g-Bifid-F	CTCCTGGAAACGGGTGG	549–563	55
	g-Bifid-R	GGTGTTCTTCCCGATATCTACA		
Bacteroides group	Bfra 531F	ATACGGAGGATCCGAGCGTTA	293	65
	Bfra 766R	CTGTTTGATACCCACACT		
Enterobacteriaceae	Eco1457F	CATTGACGTTACCCGCAGAAGAAGC	195	63
	Eco1652R	CTCTACGAGACTCAAGCTTGC		
Firmicutes	Firm_1	TGAAACTYAAAGGAATTGACG	650	60
	Firm_2	ACCATGCACCACCTGTC		

References:

Matsuki, T., Watanabe, K., Fujimoto, J., Miyamoto, Y., Takada, T., Matsumoto, K., Oyaizu, O., Tanaka, R. Development of 16S rRNA-gene-targeted group-specific primers for the detection and identification of predominant bacteria in human feces. Appl Environ Microbiol 2002, 68, 5445–51.

Malinen, E., Kassinen, A., Rinttila, T., Palva, A. Comparison of real-time PCR with SYBR Green I or 59-nuclease assays and dot-blot hybridization with rDNA-targeted oligonucleotide probes in quantification of selected faecal bacteria. Microbiology 2003, 149, 269–277.

Walter, J., Tannock, G. W., Tilsala-Timisjarvi, A., Rodtong, S., Loach, D. M., Munro, K., Alatossava, T. Detection and identification of gastrointestinal Lactobacillus species by using denaturing gradient gel electrophoresis and species-specific PCR primers. Appl. Environ. Microbiol. 2000, 66, 297–303

Vanhoutte, T., De Preter, V., De Brandt, E., Verbeke, K. Swings, J., Huys, G. Molecular Monitoring of the Fecal Microbiota of Healthy Human Subjects During Administration of Lactulose and Saccharomyces Boulardii. Appl Environ Microbiol. 2006, 72(9), 5990-7.

Bartosch, S., Fite, A., Macfarlane, G.T., McMurdo, M.E.T. Characterization of Bacterial Communities in Feces From Healthy Elderly Volunteers and Hospitalized Elderly Patients by Using Real-Time PCR and Effects of Antibiotic Treatment on the Fecal Microbiota. Appl Environ Microbiol. 2004, 70(6),3575-81.
